# A Systematic Review of Contemporary and Emerging Analgesia Techniques for Natural Labor–Patient-Centered Approaches and Technological Advances

**DOI:** 10.3390/jcm14113977

**Published:** 2025-06-05

**Authors:** Marta Bonarska, Damian Adasik, Simone Szymczyk, Gabriela Łocik, Paweł Stanirowski

**Affiliations:** 1st Department of Obstetrics and Gynecology, Medical University of Warsaw, 02-015 Warsaw, Poland; bonarskam99@gmail.com (M.B.); aadasik97@gmail.com (D.A.); gabriela.locik@gmail.com (G.Ł.); stanirowski@gmail.com (P.S.)

**Keywords:** labor anesthesia, natural delivery, multimodal analgesia, innovations in pain management

## Abstract

**Background**: Effective labor analgesia is a cornerstone of obstetric care, influencing maternal satisfaction and birth outcomes. This systematic review evaluates both conventional and emerging analgesia techniques for natural vaginal delivery, emphasizing multimodal and patient-centered strategies. **Methods**: We conducted a systematic search of PubMed, Scopus, and the Cochrane Library from January 2018 to September 2024 using MeSH terms such as “labor anesthesia”, “natural delivery”, “multimodal analgesia”, and “non-pharmacological pain management”. Randomized controlled trials, systematic reviews, meta-analyses, and cohort studies were included. Studies focusing exclusively on cesarean delivery or non-clinical interventions were excluded. The risk of bias was assessed qualitatively using the Cochrane Risk of Bias Tool for randomized trials and ROBINS-I for observational studies. However, no detailed study-by-study reporting was performed. Seventy studies met the inclusion criteria for full analysis. **Results**: Included studies were categorized into four themes: (1) neuraxial techniques (e.g., epidural, CSEA), (2) intrathecal and systemic opioids, (3) non-pharmacological approaches (e.g., TENS, hydrotherapy), and (4) technological innovations (e.g., programmed boluses, telemedicine). Neuraxial methods showed the highest analgesic efficacy and maternal satisfaction. Non-pharmacological interventions were associated with improved patient autonomy and minimal side effects. However, heterogeneity in study design and outcomes limited direct comparisons. **Limitations**: The evidence base exhibited variability in study quality, sample sizes, and reporting. The absence of standardized outcome measures, a lack of meta-analyses, and limited data on long-term outcomes limit the robustness and generalizability of the conclusions that can be drawn. **Conclusions**: This review supports a multimodal, individualized approach to labor analgesia. Future research should prioritize large, well-designed trials using standardized tools such as the VAS, PQoL, and EPDS to validate innovative techniques and ensure equitable maternal care.

## 1. Introduction

The effective management of pain during natural delivery remains a fundamental aspect of obstetric care, exerting a profound influence on maternal satisfaction, labor outcomes, and neonatal well-being. Over the decades, significant advancements have been made in anesthetic and analgesic techniques, leading to a notable diversification of options for the management of labor pain. These now include neuraxial interventions such as epidurals, as well as alternative modalities such as intrathecal opioids and intermittent boluses. Each approach presents distinctive benefits and challenges, necessitating a comprehensive understanding to ensure optimal care for women in labor. Epidural analgesia, historically regarded as the gold standard, provides effective pain relief; however, it has been associated with potential adverse effects, including prolonged labor and increased rates of instrumental delivery [1,2,3,4]. The advent of novel techniques, such as mobile epidurals and intermittent epidural boluses, has demonstrated potential in mitigating these shortcomings. A systematic review and meta-analysis demonstrated that intermittent boluses not only reduce breakthrough pain but also enhance maternal satisfaction and abbreviate labor duration in comparison to continuous epidural infusion [5]. The utilization of transversus abdominis plane blocks and other local anaesthetic techniques has been demonstrated to result in a significant reduction in the consumption of opioids and an enhancement in postoperative pain scores [6]. Furthermore, combined subarachnoid and epidural anaesthesia has demonstrated superior clinical efficacy in comparison to epidural anaesthesia alone [7,8,9]. This approach offers a faster onset and more effective muscle relaxation during labor [7,8,9]. Despite these advancements, the literature remains fragmented, with limited comprehensive evaluations that integrate both pharmacological and non-pharmacological strategies within a unified, patient-centered framework. It has been noted that emerging technologies and non-traditional approaches are often underrepresented or inconsistently reported in systematic reviews.

The objective of this systematic review is to evaluate and compare the efficacy, safety, and patient-centered outcomes of anesthetic and analgesic techniques used during natural delivery. This review also aims to identify gaps in the literature, assess emerging approaches, and highlight priorities for future research.

**Figure 1:**
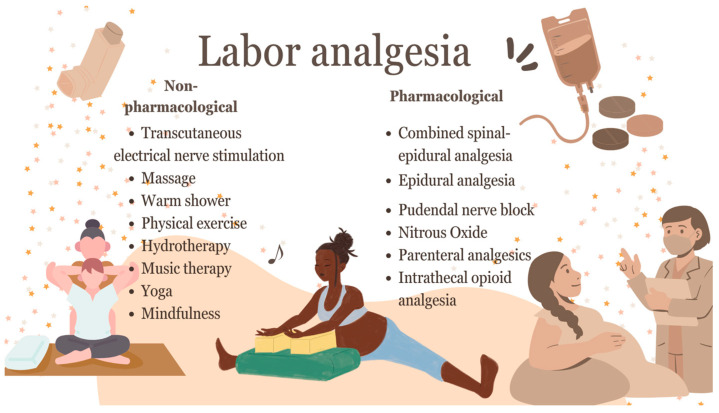
Strategies for managing labor pain.

Figure 1 provides an overview of labor analgesia strategies used in natural vaginal delivery. The figure categorizes these strategies as pharmacological or non-pharmacological, and highlights key modalities, such as neuraxial approaches, systemic analgesics, and complementary interventions.

*Pain Mechanism* *s in Labor*

**Figure 2:**
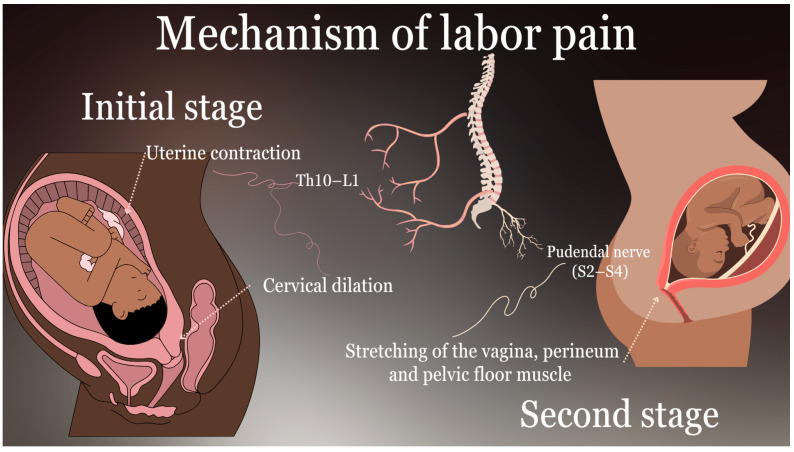
Mechanisms of labor pain.

The experience of labor pain is complex and influenced by a multitude of factors, including physiological processes and psychological factors. The intensity and nature of this pain evolve across the stages of childbirth. During the initial stage of labor, pain is primarily derived from uterine contractions and cervical dilation, which are mediated by visceral afferent nerve fibers that traverse the T10–L1 spinal segments [7,10,11,12,13]. This pain is frequently described as cramping or aching, and it is influenced by the stretching and ischemia of the uterus [7]. As labor progresses into the second stage, somatic pain becomes the predominant form of discomfort due to the descent of the fetus through the birth canal, which causes stretching of the vagina, perineum, and pelvic floor muscles [10,11]. This pain, carried by the pudendal nerve (S2–S4), is sharp and well-localized in comparison to the diffuse discomfort of the first stage [10,11]. Psychological factors, including anxiety, previous traumatic experiences, and individual pain thresholds, have a significant impact on the perception and tolerance of labor pain. These factors highlight the necessity for a comprehensive approach to labor analgesia that considers both physiological and psychological aspects. A comprehensive understanding of the underlying mechanisms of labor pain is essential for the development of effective analgesic interventions. Techniques such as combined spinal–epidural analgesia (CSEA) offer a rapid onset and effective relief for both visceral and somatic components of pain, making them a valuable option for parturients seeking comprehensive pain management during labor [9]. Addressing these mechanisms not only improves maternal comfort but also enhances the overall labor experience. The physiological mechanisms and neural pathways associated with labor pain are illustrated in Figure 2.

The accompanying diagram illustrates the neural pathways responsible for visceral and somatic pain during the first and second stages of labor, including innervation through T10–L1 and S2–S4 spinal segments.

## 2. Materials and Methods

### 2.1. Search Strategy and Study Selection

This systematic review synthesizes current and emerging techniques for labor analgesia in natural vaginal delivery. A structured search was performed in PubMed, Scopus, and the Cochrane Library, covering the literature from January 2018 to September 2024. The final search was conducted in September 2024, prior to the data extraction and screening phases. A combination of MeSH terms and keywords was employed in the search strategy, including “labor anesthesia”, “natural delivery”, “multimodal analgesia”, “non-pharmacological pain management”, “neuraxial techniques”, and “innovations in labor pain relief”. The utilization of Boolean operators “AND” and “OR” was employed to optimize the precision of the search. Subsequently, all results were imported into EndNote for reference management, and duplicates were automatically removed. Two reviewers independently screened titles and abstracts using predefined inclusion criteria. Eligible studies were those that focused on labor analgesia during vaginal delivery and reported outcomes such as pain relief, maternal satisfaction, or neonatal safety.

Eligibility Criteria:

Studies were eligible for inclusion if they were published in peer-reviewed English-language journals between January 2018 and September 2024, involved human subjects undergoing natural vaginal delivery, and assessed the effects of anesthetic or analgesic interventions on outcomes such as pain intensity, maternal satisfaction, delivery duration, or neonatal condition. Eligible study designs included randomized controlled trials, cohort studies, case-control studies, and systematic reviews or meta-analyses.

Studies were excluded if they focused exclusively on cesarean sections or postpartum pain management, involved animal or preclinical models, were case reports, conference abstracts, or unpublished gray literature, or lacked original outcome data (e.g., theoretical reviews or narrative articles without empirical results).

**Figure 3:**
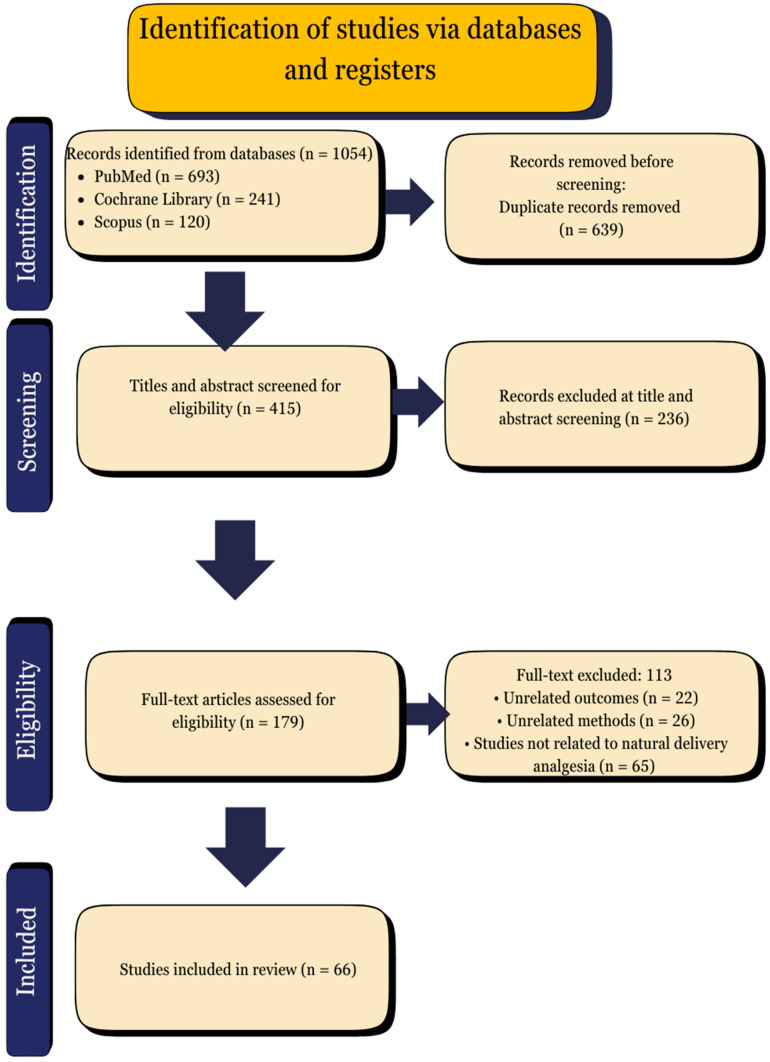
PRISMA flow diagram.

The full-text articles were meticulously assessed, and any discrepancies were resolved through discussion or consultation with a third reviewer. The selection process is illustrated in the PRISMA flow diagram (Figure 3). The reviewed studies are presented in Table S1.

### 2.2. Data Extraction and Analysis

The process of data extraction was executed independently by two reviewers who utilized a standardized form for the collection of pertinent information. The information collected encompassed a range of domains, including the study design, the sample size, the population characteristics, the intervention details, the comparators, the outcomes, and the limitations. The primary outcomes of interest included the effectiveness of pain relief, maternal satisfaction, labor duration, mode of delivery, and neonatal safety. Secondary data items included demographic details, intervention techniques, and funding sources. Any missing or unclear information was noted and addressed during the review process. The risk of bias was assessed using the Cochrane Risk of Bias Tool for randomized trials and ROBINS-I for non-randomized studies, with two reviewers working independently. Given the clinical and methodological diversity of the included studies, a narrative synthesis approach was employed. The studies were grouped into four thematic categories: neuraxial techniques, multimodal strategies, non-pharmacological methods, and technological innovations. Due to substantial clinical and methodological heterogeneity across the included studies, including variability in intervention protocols, outcome definitions, measurement scales, and timepoints, quantitative meta-analysis was not performed. Therefore, a narrative synthesis was adopted to summarize and interpret the findings while minimizing the risk of introducing bias through inappropriate data pooling.

The management of missing data was conducted through a descriptive approach, and the execution of subgroup or sensitivity analyses was not undertaken.

### 2.3. Outcomes

The outcomes were analyzed and reported according to the thematic grouping of interventions. The findings of each study were summarized in terms of pain reduction, maternal satisfaction, neonatal outcomes, and any reported adverse effects. However, given the limitations imposed by differences in study design, population, and intervention protocols, direct comparisons were not feasible. Consequently, the calculation of pooled effect sizes was not possible. Instead, the results were described in narrative form to highlight patterns, trends, and gaps in the evidence. The comprehensive summary of the included studies is presented in Table S1, and the methodological limitations are addressed in the Discussion section. Language editing and PRISMA guideline alignment support were provided with the assistance of ChatGPT 4.0. The following PRISMA flow diagram illustrates the study selection process for the systematic review. This encompasses the quantity of records that have been identified, screened, assessed for eligibility, and ultimately incorporated into the final synthesis.

## 3. Results

### 3.1. Epidural Analgesia

Epidural analgesia remains the gold standard for labor pain management, due to its high efficacy and favorable safety profile. The administration of a combination of local anesthetics and opioids into the epidural space results in the effective blockade of pain transmission from T10 to L1 during the first stage of labor and from S2 to S4 during the second stage [11]. A review of the literature reveals that 85–90% of women who receive epidural analgesia report significant pain relief during labor, a rate that is considerably superior to that achieved with systemic opioids or non-pharmacological methods, which provide adequate pain control in only 50–60% of cases [14,15]. Advances in epidural analgesia, including the introduction of low-dose regimens that combine reduced concentrations of local anesthetics with opioids, have resulted in improved maternal mobility, a reduction in the incidence of motor block by over 40%, and a decrease in the occurrence of complications such as hypotension [11,16,17]. Nevertheless, epidural analgesia does have an impact on labor dynamics. Epidural analgesia has been demonstrated to be associated with a lengthier second stage of labor and an augmented relative risk of instrumental vaginal delivery, with a reported relative risk of 1.44 (95% CI: 1.29 to 1.60) [18]. In contrast, Olszynska et al. reported that patients who received epidural analgesia experienced significantly longer labor (415 vs. 255 min) and higher rates of instrumental delivery compared to those who did not receive epidural analgesia, although the rate of cesarean delivery was lower [19]. In a study conducted by Shumeli, it was determined that the use of epidurals resulted in an average increase of 82 min in the second stage of labor for both nulliparous and multiparous subjects [20]. A novel approach to epidural delivery, programmed intermittent epidural boluses (PIEBs), has been demonstrated to enhance analgesic spread, reduce local anesthetic consumption by 20% and improve spontaneous vaginal delivery rates in comparison to continuous infusion [5,16,21]. From a patient-centered perspective, epidural analgesia is highly valued for its effectiveness in eliminating labor pain while allowing the mother to remain conscious and engaged in the birthing process [16]. A review of the literature revealed that maternal satisfaction with epidural analgesia reached 96.6%, a figure that is significantly higher than that reported for alternative methods such as transcutaneous electrical nerve stimulation (TENS) [22]. Fernandes et al. revealed that 87% of the female participants rated their overall birth experience as positive, with 96% of them receiving labor epidural analgesia and 82.1% of them rating the analgesia as highly effective [23]. Nevertheless, epidural analgesia is not without its own set of potential complications. These include transient fetal bradycardia, which occurs in approximately 20% of cases, maternal pruritus (15–20%), and rare complications such as post-dural puncture headache (1–3%) [12,24]. These manageable risks underscore the importance of individualized care and informed consent. As a cornerstone of labor analgesia, epidural analgesia continues to evolve with innovations aimed at optimizing safety, maintaining labor dynamics, and achieving unparalleled efficacy in pain management.

### 3.2. Intrathecal Opioid Analgesia

Intrathecal opioid analgesia represents a highly efficacious approach for the management of labor pain, distinguished by its rapid onset and minimal motor blockade. This technique entails the administration of a single injection of lipophilic opioids, such as fentanyl or sufentanil, into the subarachnoid space, thereby providing targeted analgesia for visceral pain associated with cervical dilation [25]. The results of the study indicated that intrathecal sufentanil provided analgesia with a mean onset time of 5.93 ± 2.87 min and an average reduction in pain scores to 1.08 ± 0.16 [26]. The neonatal outcomes, including the Apgar scores, remained unaltered, and pruritus was the most prevalent adverse effect [26]. The duration of analgesia provided by sufentanil was 113 ± 45 min [27]. Conversely, fentanyl achieved notable pain relief within 3.6 ± 2.1 min, with a duration of approximately 103 ± 22 min [27]. This method is particularly efficacious in the context of early labor or as an adjunct to combined spinal–epidural techniques, offering rapid and effective pain control while preserving maternal mobility [28,29]. In contrast to continuous epidural analgesia, intrathecal opioids exert a negligible influence on labor dynamics [30]. The available evidence indicates that this technique does not prolong the duration of labor stages or increase the likelihood of instrumental deliveries, making it a suitable choice for patients who prefer a low-intervention approach [5,30]. Furthermore, patient-centered outcomes consistently indicate high satisfaction with intrathecal opioid analgesia, with satisfaction rates exceeding 85%. This is attributed to the rapid efficacy of the technique and its maintenance of maternal autonomy during labor [26,28,29]. Nevertheless, the technique is not without potential adverse effects. The incidence of pruritus was 42.6% among patients who received intrathecal opioids, while the incidence of nausea and vomiting was 13.1% [31,32]. While these side effects are bothersome, they are typically self-limiting and do not significantly impact overall satisfaction [12]. In rare instances, higher doses of opioids may result in transient fetal bradycardia, necessitating close monitoring [26,31,33]. Notwithstanding these limitations, intrathecal opioid analgesia continues to represent a valuable tool for the management of labor pain, particularly in settings where rapid onset and minimal motor block are of primary importance. By balancing its efficacy and safety profile, this technique offers a patient-centered approach that aligns with diverse clinical scenarios.

### 3.3. CSEA

CSEA represents a highly efficacious technique for the management of labor pain. It integrates the rapid onset of spinal analgesia with the flexibility of continuous epidural infusion, thereby providing prolonged pain relief [28,31]. This approach provides substantial pain relief within a timeframe of 2–5 min, with over 90% of parturients achieving adequate analgesia during the initial stages of labor [28,33]. The dual-action mechanism permits the administration of lower doses of anesthetics and opioids, thereby minimizing the incidence of motor block while maintaining effective pain control [8,28,34]. Nevertheless, intrathecal opioids in the spinal component may occasionally result in transient adverse effects, including pruritus, which affects 42.6% of patients, and transient fetal bradycardia in up to 2.38% of cases [31]. The impact of CSEA on labor dynamics has been the subject of research, with findings indicating that it does not markedly prolong labor stages in comparison to traditional epidural analgesia [34,35]. In contrast, an alternative study indicates that CSEA resulted in a shorter duration for the initial stage of labor (218.93 ± 78.15 min) compared to epidural analgesia (308.03 ± 147 min) [36]. No significant difference was observed in the duration of the second stage of labor between the two groups [36]. Moreover, maternal satisfaction rates exceed 85%, driven by the immediate onset of pain relief and the ability to participate actively in labor [26,35,37]. In a study of parturients who received CSEA, 97% reported effective labor analgesia, as evidenced by a Visual Analog Scale (VAS) pain score of 0 [34]. Adverse effects and trade-offs associated with CSEA include transient maternal hypotension, pruritus, and, in rare cases, post-dural puncture headache [31,34,38,39]. The flexibility of the epidural catheter reduces the need for repeat interventions, enhancing safety and reducing complications [31]. With appropriate monitoring and dose adjustments, CSEA remains a cornerstone of neuraxial analgesia, offering a balanced approach that aligns with patient preferences and clinical requirements.

### 3.4. Pudendal Nerve Blocks

A pudendal nerve block is a regional anesthetic technique that is commonly utilized in the second stage of labor to provide effective perineal analgesia, particularly during instrumental deliveries or episiotomy [40]. This technique has been demonstrated to be highly efficacious, with a significant reduction in pain scores during perineal suturing and delivery. Studies have shown that a pudendal nerve block significantly reduces pain scores (VAS) in the first 6 h postpartum in comparison to local infiltration [40]. The minimal impact on motor function and lack of systemic drug absorption make it a safer alternative for patients with contraindications to epidural or spinal analgesia [41]. A pudendal nerve block has been demonstrated to have a negligible impact on labor dynamics, as it does not interfere with uterine contractions or prolong labor stages [41,42]. Xu et al. showed that a pudendal nerve block shortened the second stage of labor by 33.8 min without affecting labor progress or neonatal outcomes [41]. However, it is primarily utilized during the second stage of labor and may not provide sufficient analgesia for earlier stages [41,43]. This is because the pudendal nerve is primarily responsible for somatic pain in the second stage of labor, making PNB appropriate for this stage, but ineffective for visceral pain emanating from the uterus and cervix in the first stage [11]. Patient satisfaction scores have been consistently high, reflecting the minimal discomfort associated with the procedure [40,41]. Adverse effects are uncommon but may include transient paresthesia, a local hematoma, and, in rare cases, pudendal nerve injury [44,45]. The utilization of ultrasound guidance has resulted in a reduction in the incidence of complications, with the incidence of hematoma [46]. However, the efficacy of this technique is contingent upon the accurate identification of the pudendal nerve, which necessitates technical expertise and training.

### 3.5. Nitrous Oxide (Inhalational Analgesia)

Nitrous oxide (NO) is a commonly utilized, non-invasive method of labor analgesia that provides moderate pain relief with a favorable safety profile for both the mother and neonate. Although its analgesic efficacy is inferior to that of neuraxial techniques such as epidurals, studies have demonstrated that 31% of women who utilize NO during labor do not necessitate a transition to more invasive analgesics [47]. Another study demonstrated that 40–60% of women who utilized NO ultimately transitioned to epidural analgesia [48]. In contrast, McGarrigle et al. investigated patient preferences for NO and found that while some women opted for epidural analgesia, a notable proportion valued the autonomy and rapid pain relief afforded by NO [49]. A randomized study demonstrated that nitrous oxide significantly reduced VAS scores in comparison to systemic opioids, such as pethidine (*p* = 0.0001) [50]. The rapid onset of action and ease of self-administration are particularly valued, as they enable patients to maintain autonomy over pain management [51]. Nitrous oxide has a minimal impact on labor dynamics, preserving mobility and avoiding interference with uterine contractions, which is a distinct advantage over systemic opioids or epidurals [47,52,53]. Maternal satisfaction rates with NO are consistently high, often attributed to its ability to enhance relaxation, reduce anxiety, and align with patients’ preferences for less invasive options [53]. Approximately 50–75% of women who used nitrous oxide during labor reported being satisfied with their pain relief experience [51]. However, adverse effects such as dizziness, nausea, and vomiting are reported in approximately 8% of cases, and long-term occupational exposure poses potential risks to healthcare staff [54,55,56].

### 3.6. Parenteral Analgesics (Systemic Opioids)

Parenteral analgesics, including opioids such as pethidine and tramadol, are commonly utilized for the management of labor pain due to their ease of administration and relatively low cost, particularly in settings with limited access to neuraxial techniques. Although opioids are effective in reducing pain, their analgesic efficacy is moderate, with up to 40% of women requiring additional analgesia or conversion to neuraxial methods such as epidurals [57,58]. Their impact on labor progression is minimal. Meher et al. showed that systemic opioids (pethidine and phenargon) did not prolong labor compared to epidural analgesia [59].

However, parenteral analgesics are associated with maternal drowsiness and transient nausea or vomiting, which occurs in approximately 20% of cases [58,60]. The potential risk of respiratory depression in neonates was identified as a potential consequence of opioid use [18,58,61,62]. In the study by Habib et al., newborns exposed to meperidine exhibited significantly diminished 1 min Apgar scores relative to those who received paracetamol [60]. Nevertheless, no discernible differences were identified in 5 min Apgar scores or the incidence of neonatal respiratory distress [60]. In contrast, an alternative study demonstrated no statistically significant differences in mean Apgar scores or incidence of neonatal complications between neonates whose mothers received intravenous remifentanil or epidural analgesia during labor [63]. The potential risks of neonatal respiratory depression associated with the use of systemic opioids during labor highlight the necessity for the exploration of alternative techniques for pain management [61].

### 3.7. Non-Pharmacological Methods

Non-pharmacological methods for labor pain management are increasingly recognized for their effectiveness in reducing pain intensity, enhancing maternal satisfaction, and minimizing adverse effects [64,65]. A variety of techniques, including massage, hydrotherapy, TENS, and relaxation practices, have demonstrated encouraging outcomes across a range of metrics. A series of non-pharmacological interventions, including massage, warm showers, and exercise, have been shown to reduce labor pain scores by 24 mm on the VAS and delay the necessity of pharmacological analgesia by approximately 50 min [66]. Concurrently, these interventions have been observed to shorten the total duration of labor by an average of 18 min [66].

A study by Ergin et al. revealed that hydrotherapy led to a VAS pain score reduction of approximately 20 mm during the initial stage of labor when compared to standard care [67]. Additionally, the study found that hydrotherapy was associated with a labor duration reduction of 32 min [67].

A recent study that incorporated a combination of deep breathing, relaxation techniques, and counter-pressure massage reported significant reductions in labor pain. The study’s findings, which included *p*-values of 0.002 for deep breathing and 0.046 for massage, confirm that these interventions are statistically effective in reducing labor pain intensity [68].

A meta-analysis of over 6000 women revealed that TENS, music therapy, and acupressure were associated with a significant pain reduction (average VAS score decrease by 15–20 mm) during the first stage of labor and higher satisfaction rates (around 85%) [65]. Recent studies have demonstrated that relaxation techniques, such as yoga and massage, can result in a quantifiable reduction in labor pain intensity. Specifically, yoga has been shown to reduce pain intensity by approximately 6.1 points on the VAS scale, while massage has been found to reduce pain intensity by 1.9 points. Additionally, these techniques have been observed to enhance maternal satisfaction and reduce anxiety levels during childbirth [69,70].

The overall rate of adverse effects for non-pharmacological methods remains low, generally under 3% for mild complications such as dizziness or transient discomfort [71]. Nevertheless, the efficacy of these techniques is contingent upon appropriate implementation and alignment with patient preferences.

### 3.8. Challenges and Barriers to Implementation

The implementation of labor analgesia in natural delivery is confronted by numerous barriers, including resource limitations, cultural perceptions, and systemic challenges. In settings with limited resources, the dearth of trained personnel, epidural equipment, and infrastructure severely curtails access to effective analgesia. For instance, a survey conducted in Egyptian maternity units revealed that 44.9% of laboring women relied exclusively on non-pharmacological methods due to limited resources, despite the high demand for analgesia [72]. In higher-income regions, systemic factors, including reimbursement policies and misconceptions about safety, further hinder widespread adoption. A study in Japan revealed that only 6.1% of parturients received labor analgesia, compared to 70% in the United States, due to cultural biases and concerns about maternal safety [73]. Furthermore, inadequate prenatal education on pain management options often results in inadequate patient advocacy for analgesia. Misconceptions about labor analgesia, such as its impact on delivery outcomes, persist among healthcare providers and patients alike, further delaying adoption [74]. Addressing these barriers necessitates investments in training, infrastructure, and community education to ensure equitable access to labor analgesia.

### 3.9. Future Directions in Labor Analgesia

Innovations in labor analgesia are increasingly focusing on patient-centered approaches, enhanced safety, and technological integration. Advancements in neuraxial techniques, including dural puncture epidurals and CSEA, are aimed at optimizing pain relief while minimizing motor block, thereby preserving maternal mobility during labor [75]. Emerging technologies, including closed-loop systems for automated drug delivery, hold considerable potential in delivering precise analgesic doses tailored to individual needs, thereby reducing complications associated with human error [76]. Furthermore, multimodal analgesic strategies, encompassing both pharmacological and non-pharmacological methods, such as acupuncture and hydrotherapy, are gaining traction for their holistic benefits and ability to meet diverse patient preferences [73]. Furthermore, the integration of remote monitoring and telemedicine into labor management systems is a subject of ongoing exploration, with the aim of enhancing accessibility and continuity of care, particularly in underserved regions [77].

## 4. Discussion

This systematic review offers a comprehensive synthesis of pharmacological and non-pharmacological methods for labor analgesia, with findings that broadly align with the current body of literature. Neuraxial techniques, particularly epidural analgesia and CSEA, have been demonstrated to consistently provide the most effective pain relief and the highest level of maternal satisfaction across studies. However, epidural analgesia was associated with moderate prolongation of the second stage of labor and increased rates of instrumental delivery. Conversely, CSEA demonstrated a more favorable profile regarding labor progression. In contrast, the administration of intrathecal opioid analgesia resulted in a swift onset of pain relief and preservation of maternal mobility. However, its analgesic efficacy was found to be somewhat diminished, and its administration necessitated meticulous management of pruritus and nausea. Pudendal nerve blocks emerged as an effective option for perineal analgesia in the second stage of labor, offering high efficacy with negligible motor impairment. However, these blocks lacked effectiveness for visceral pain during the initial phase of labor.

Non-pharmacological methods, including massage, hydrotherapy, and TENS, have been shown to provide moderate analgesia while promoting maternal autonomy, relaxation, and satisfaction, with no associated pharmacologic adverse effects. Nitrous oxide has been demonstrated to provide flexible, rapid-onset analgesia with minimal impact on labor progression; however, its efficacy in pain control is comparatively inferior to neuraxial methods. Consequently, the selection of an analgesic strategy should be tailored to the individual patient’s clinical circumstances, the phase of labor, the availability of resources, and patient preference. This approach entails a judicious balancing of the superior analgesia associated with neuraxial methods against the benefits of greater mobility, autonomy, and fewer interventions that are characteristic of non-pharmacologic and systemic options. Despite the robustness of the findings across a range of interventions, the evidence base is not without limitations. A significant number of the included studies demonstrated substantial heterogeneity in their design, population characteristics, and outcome measures, which complicates the establishment of direct comparisons. Some trials exhibited small sample sizes or lacked blinding, which can potentially introduce bias. While the risk of bias was qualitatively assessed using the Cochrane Risk of Bias Tool and ROBINS-I, a formal study-by-study evaluation was not systematically reported, which may affect the interpretability of individual study findings. Furthermore, satisfaction and safety outcomes are frequently derived from self-reported measures, which may be influenced by subjective factors and cultural context.

It is imperative to acknowledge that the applicability and selection of analgesia methods are influenced by patient-specific factors. Pregnancies that are considered high risk, including those complicated by coagulopathies, preeclampsia, or cardiovascular disorders, may contraindicate the utilization of neuraxial techniques due to safety concerns. Conversely, women with a strong preference for natural childbirth, or those with prior negative experiences with pharmacologic interventions, may favor non-pharmacological methods. A multitude of additional factors, including anxiety levels, cultural beliefs, body mass index, and provider availability, also exert influence on the decision-making process. These factors underscore the imperative for shared decision-making processes and customized analgesia strategies, contingent on clinical appropriateness and patient values.

The review process itself was constrained by several factors. Firstly, the restriction to English-only studies limited the scope of the review. Secondly, the exclusion of gray literature limited the comprehensiveness of the review. Thirdly, the absence of meta-analysis, owing to the substantial clinical and methodological heterogeneity across studies, limited the ability to produce pooled effect estimates and draw definitive conclusions. Despite the comprehensive nature of the search strategy, the utilization of automation tools and the manual extraction of data may compromise the reproducibility of results.

The quality of the evidence supporting neuraxial techniques was generally higher, with multiple large randomized controlled trials (RCTs) and systematic reviews providing robust data. Conversely, the evidence supporting non-pharmacological methods and systemic opioids frequently stems from smaller trials that are susceptible to greater risk of bias, inconsistent outcome reporting, and heterogeneous intervention protocols. These discrepancies in the quality of the evidence necessitate a cautious interpretation of the non-pharmacological findings and underscore the necessity for more standardized, high-quality trials in this domain.

The findings of this study have practical implications for clinical practice and policy. They advocate for a flexible, patient-centered approach to labor analgesia that balances analgesic efficacy, safety, maternal satisfaction, and individualized clinical circumstances. Future research should prioritize high-quality, large-sample randomized trials comparing emerging techniques (e.g., programmed intermittent epidural bolus, telemonitored analgesia) across diverse settings. Longitudinal studies examining maternal–neonatal outcomes and quality of life are also needed to guide evidence-based improvements in labor pain management. Future research endeavors should prioritize the execution of high-quality, large-sample randomized trials that methodically compare emerging techniques across a diverse array of settings. In order to comprehensively assess maternal well-being, studies should employ validated measures such as the Perinatal Quality of Life (PQoL) scale and the Edinburgh Postnatal Depression Scale (EPDS).

The identification and resolution of barriers to the implementation of labor analgesia necessitate a comprehensive and multifaceted approach. The expansion of access to training for healthcare providers, the enhancement of infrastructure for neuraxial techniques in settings with limited resources, and the augmentation of antenatal education for pregnant women regarding available pain relief options have the potential to substantially increase uptake. Furthermore, culturally sensitive approaches and policy reforms aimed at integrating labor analgesia into standard maternal care guidelines have the potential to reduce misconceptions and enhance equitable access across diverse populations.

Addressing these research gaps is imperative for the advancement of the field of labor analgesia, ensuring that innovative, patient-centered care is both effective and equitable across global populations.

## 5. Conclusions

### 5.1. Epidural Analgesia

Epidural analgesia has been shown to provide significant pain relief for 85–90% of laboring women, outperforming systemic opioids and non-pharmacological methods, which achieve only 50–60% efficacy [14,15,18,78]. Moreover, the satisfaction rates among mothers who received epidural analgesia during childbirth reached up to 96.6%, with 87% of women expressing a positive opinion of their birth experience [5,16,23]. Innovations such as low-dose regimens and programmed intermittent epidural boluses (PIEBs) have led to a reduction in motor block by over 40% and local anesthetic use by 20% [5,16]. However, epidurals have been observed to increase the duration of the second stage of labor [18,20]. Common side effects include transient fetal bradycardia (20%), maternal pruritus (15–20%), and post-dural puncture headache (1–3%) [12,33].

### 5.2. Intrathecal Opioid Analgesia

Intrathecal opioids have been demonstrated to provide rapid analgesia, with an average onset time ranging from 3.6 to 5.9 min, and a reduction in pain scores to 1.08 ± 0.16 [26,27]. The duration of pain relief ranges from 103 to 113 min [26,27]. Satisfaction rates exceed 85%, with minimal impact on labor progression or delivery mode [26,28,30]. Adverse effects include pruritus (42.6%), nausea/vomiting (13.1%), and rare instances of transient fetal bradycardia [26,31,33]. Notably, these adverse effects do not appear to compromise neonatal Apgar scores, thereby underscoring the safety profile of the technique [26].

### 5.3. CSEA

CSEA has been shown to achieve analgesia within 2–5 min and has been found to provide effective pain relief in over 90% of patients [28]. A study reported VAS scores of 0 in 97% of women receiving CSEA [36]. Satisfaction rates consistently exceeded 85%, and the method allows for active participation in labor due to preserved mobility [35,37]. Labor duration may be reduced in some cases; for instance, the first stage was shortened to 218.93 ± 78.15 min with CSEA, compared to 308.03 ± 147 min with standard epidurals [36]. Adverse effects include pruritus (42.6%), hypotension, and rare cases of fetal bradycardia (~2.38%) [31].

### 5.4. Pudendal Nerve Block

Guided by ultrasound, pudendal nerve blocks have been shown to achieve a success rate of over 90% in alleviating perineal discomfort, particularly in contexts involving instrumental delivery or episiotomy [79]. One study demonstrated a 33.8 min reduction in the second stage of labor [41]. A significant reduction in VAS pain scores was observed during the first 6 h postpartum in comparison to local infiltration [40]. Patient satisfaction remains consistently high [40,41]. Adverse effects, if present, are transient and include paresthesia, local hematoma, and pudendal nerve injury. It is noteworthy that complication rates are reduced through ultrasound guidance [44,45].

### 5.5. NO

Nitrous oxide has been found to be moderately effective, with 31% of women not requiring transition to more invasive methods. However, 40–60% eventually opted for epidurals [47,48]. The level of maternal satisfaction is notably high, primarily attributable to the autonomy and swift onset characteristics of the method [47,49]. The method preserves mobility and does not affect labor dynamics [52,53]. Adverse effects, including but not limited to nausea, dizziness, and vomiting, occurred in approximately 8% of cases [54]. Concerns regarding long-term occupational exposure risks for staff persist [56].

### 5.6. Systemic Opioids

Systemic opioids have been demonstrated to provide moderate pain relief; however, in up to 40% of cases, additional analgesia may be required [18,58]. Maternal drowsiness and nausea/vomiting occurred in approximately 20% of patients [58,60]. Neonatal respiratory depression is a significant concern; meperidine, for instance, was associated with lower 1 min Apgar scores compared to paracetamol [60]. However, 5 min scores and neonatal distress rates generally remained unaffected [60]. Some studies have found no significant differences in neonatal outcomes between systemic opioids and epidural use [63].

### 5.7. Non-Pharmacological Methods

A growing body of research has demonstrated the efficacy of non-pharmacological interventions, such as massage, hydrotherapy, TENS, and relaxation techniques, in reducing pain scores on the VAS scale by up to 24 mm. These approaches have also been shown to delay the administration of pharmacologic analgesics [66]. A randomized trial also demonstrated an 18 min reduction in labor duration with sequential non-pharmacological interventions [66]. A comprehensive meta-analysis of 63 studies revealed a significant reduction in pain during the initial stage of labor, accompanied by enhanced maternal satisfaction and a low incidence of adverse effects [65]. The application of deep breathing relaxation techniques in conjunction with massage therapy has been demonstrated to effectively mitigate labor pain. [68].

### 5.8. Summary

This review confirms that neuraxial techniques continue to offer the most effective pain relief during labor, while alternative and non-pharmacological methods provide promising, patient-centered options. Among these, epidural and combined spinal–epidural analgesia are supported by the strongest evidence base, including multiple randomized controlled trials and systematic reviews demonstrating high analgesic efficacy, maternal satisfaction, and acceptable safety profiles. Intrathecal opioids and pudendal nerve blocks also represent evidence-based, targeted options with distinct advantages in specific labor stages or clinical scenarios. Non-pharmacological interventions, despite their comparatively diminished potency in terms of analgesic strength, assume an indispensable role within the framework of holistic, minimally invasive care. These interventions hold particular value in the context of early labor, as well as circumstances wherein neuraxial options are deemed contraindicated.

An integrated, individualized approach—combining pharmacological and non-pharmacological techniques—emerges as the most adaptable strategy, aligning with the evolving emphasis on maternal autonomy, mobility, and informed choice. The clinical implementation of these guidelines should prioritize methods with robust evidence, while also tailoring analgesia plans to institutional resources, patient preferences, and obstetric indications.

In the future, research endeavors should prioritize the validation of emerging techniques and technologies across diverse populations. These efforts should employ standardized outcome measures that encompass both the clinical and psychosocial dimensions of maternal well-being. This approach is imperative to guarantee that labor analgesia advances towards safer, more effective, and more equitable care on a global scale.

A comparative summary of the efficacy, maternal satisfaction, labor impact, neonatal outcomes, and adverse effects across analgesia techniques is presented in Table 1.

## Figures and Tables

**Table 1:** A comparative summary of the efficacy, maternal satisfaction, labor impact, neo-natal outcomes, and adverse effects across analgesia techniques.

Technique	Pain Relief Efficacy	Maternal Satisfaction	Labor Impact	Neonatal Outcomes	Adverse Effects
Epidural Analgesia	85–90% of women report significant relief	Very high (up to 96.6%)	Prolongs 2nd stage by 16–82 min; 10–15% ↑ instrumental delivery	No major adverse effects	Pruritus (15–20%), transient fetal bradycardia (20%), post-dural puncture headache (1–3%)
CSEA	Rapid onset; >90% effective	High (>85%)	Shortens 1st stage; no significant impact on 2nd stage	No significant differences in Apgar scores	Pruritus (42.6%), hypotension, rare post-dural headache
Intrathecal Opioids	Fast onset (3.6–5.9 min); pain scores ↓ to ~1.08	High (>85%)	Minimal impact on labor dynamics	No significant Apgar score effects	Pruritus (42.6%), nausea (13.1%), rare transient fetal bradycardia
Pudendal Nerve Block	Highly effective for perineal pain (success rate >90%)	Consistently high	Shortens 2nd stage by ~33.8 min	No adverse neonatal impact	Rare paresthesia, hematoma, nerve injury (↓ with ultrasound guidance)
Nitrous Oxide (NO)	Moderate; ~31% avoid escalation to neuraxial methods	Moderate (50–75%)	Preserves mobility; no prolongation of labor	Favorable safety profile	Nausea, dizziness, vomiting (~8%); staff exposure risk
Systemic Opioids (e.g., pethidine, tramadol)	Moderate; up to 40% require additional analgesia	Moderate	Minimal impact on labor duration	Possible ↓ 1 min Apgar; generally safe at 5 min	Drowsiness (20%), nausea, risk of neonatal respiratory depression
Non-Pharmacological Methods (massage, TENS, hydrotherapy, relaxation)	Mild to moderate (VAS ↓ by 12–24 mm)	High (85–88%)	Shortens labor by 18–32 min	Safe	Rare mild adverse effects (<3%) such as dizziness

## Data Availability

The data presented in this study are available on request from the corresponding author. The data are not publicly available due to privacy restrictions.

## Supplementary Materials

The following supporting information can be downloaded at: https://www.mdpi.com/article/10.3390/jcm14113977/s1, Table S1: Summary of the reviewed literature.
